# Optical coherence tomography and electrophysiology of retinal and visual pathways in Wilson’s disease

**DOI:** 10.1007/s11011-015-9776-8

**Published:** 2015-12-21

**Authors:** Ewa Langwińska-Wośko, Tomasz Litwin, Kamil Szulborski, Anna Członkowska

**Affiliations:** Department of Ophthalmology, Medical University of Warsaw, Warsaw, Poland; 2nd Department of Neurology, Institute of Psychiatry and Neurology, Sobieskiego 9, 02-957 Warsaw, Poland; SPKSO Ophthalmic University Hospital, Medical University of Warsaw, Warsaw, Poland; Department of Experimental and Clinical Pharmacology, Medical University of Warsaw, Warsaw, Poland

**Keywords:** Wilson’s disease, Brain magnetic resonance, Optical coherence tomography, Pattern-reversal visual evoked potentials, Electroretinography

## Abstract

We evaluated correlations between positive findings of changes on brain magnetic resonance imaging (MRI) and selected morphological and electrophysiological parameters of the retinal and visual systems in Wilson’s disease. Fifty-eight Wilson’s disease symptomatic patients were divided according to whether they displayed brain changes on MRI (positive, *n* = 39; negative, *n* = 19). All participants and healthy control group (*n* = 30), underwent retinal optical coherence tomography to assess the thickness of macula and the total retinal nerve fiber layer. Visual evoked potentials were measured and electroretinography was performed. Macular and retinal nerve fibers were thinner in participants with changes on MRI than in participants without changes. Electrophysiological parameters were markedly different in the MRI positive group compared with the negative group and 30 healthy controls; however, some abnormalities were evident in cases without visible brain pathology. Morphological and electrophysiological changes of retinal and visual pathways are associated with MRI visualized brain injury in Wilson’s disease and may be useful for detecting the degree of neurodegeneration.

## Introduction

Copper is an essential trace element. However, due to its toxicity at higher concentrations, its level in the body must be very strictly controlled (Valentine and Gralla 1997). Wilson’s disease (WD) is an autosomal recessive genetic disorder caused by mutations in the gene encoding the ATP7B protein, which is responsible for copper transport and excretion (Bull et al. 1993). As a result of defective protein production, excess copper accumulates in the body, leading to damage to tissues and organs; this damage occurs predominantly in the liver (Ferenci 2005), and later becomes evident in the central nervous system, cornea, and other organs (Scheinberg and Sternlieb 1996; Litwin et al. 2013a). Therefore, the typical clinical signs and symptoms of WD are hepatic, neuropsychiatric, and ophthalmological such as the Kayser-Fleischer ring in the cornea (Scheinberg and Sternlieb 1984; Roberts and Schilsky 2008; EASL Clinical Practice Guidelines 2012).

Currently, the most common investigations used to aid WD diagnosis are measurements of serum ceruloplasmin and copper concentrations, 24-h urinary copper excretion, as well as DNA analysis, liver-function tests, detection of Kayser-Fleischer rings, and magnetic resonance imaging (MRI) of the brain (Ala et al. 2007). MRI also enables the detection of brain pathology that develops in later stages of the disease (Bandmann et al. 2015). Changes usually occur in the basal ganglia (putamen, globus pallidus), thalamus, and pons and appear in most WD patients with the neuropsychiatric form (90–100 %) of the disease (Litwin et al. 2013b), but copper accumulates in all parts of brain (Scheinberg and Sternlieb 1984; Litwin et al. 2013a). Brain changes are less common in the hepatic form (40–50 %) and rare in asymptomatic cases (25 %) (Litwin et al. 2013a, b).

The embryological, structural, and functional continuity of the retina with the central nervous system makes the visual pathway a prime target for potential non-invasive investigations of neurodegeneration, such as the neurodegeneration that occurs in the course of Alzheimer’s disease and multiple sclerosis (Greenberg and Frohman 2010). Modern techniques detect even very subtle changes in retinal and proximal visual pathways. Morphological abnormalities of the retina can be detected using optical coherence tomography (OCT), a non-invasive imaging technique that utilizes illumination with optical beams in a manner analogous to the mechanical waves of an ultrasound scanner (Gramatikov 2014). The relatively low wavelength of visible light paired with interference-based noise filtering yields crisp, high-resolution images. Functional assessment of the retina can be performed with established electrophysiological techniques such as testing of visual evoked potentials (Halliday 1993) and electroretinography (ERG). Retinal copper deposition should affect the conduction properties of the retina, which is an extension of the central nervous system (Satishchandra and Ravishankar Naik 2000).

Although cortical accumulation of copper in the cornea is typical for WD, retinal involvement in WD has not been widely investigated especially taking into account various stages of the disease (Satishchandra, et al. 2000; Albrecht et al. 2012). The aims of the present study were to assess the morphological and functional status of retinal and visual pathway as markers of neurodegeneration, to relate this status to the presence of lesions on brain MRI in a large cohort of WD patients, and to identify ophthalmological markers of WD.

## Methods

This study was a cross-sectional, non-interventional, observational study approved by the Medical University of Warsaw Bioethics Committee. All participants provided informed consent. All consecutive symptomatic WD patients admitted to the 2nd Department of Neurology, Institute of Psychiatry and Neurology, Warsaw between 2012 and 2013 who were diagnosed according to established criteria (EASL Clinical Practice Guidelines 2012) were enrolled in the study. The disease was considered symptomatic if a patient had clinical signs of WD at the time of diagnosis or earlier. We distinguished between neurological and hepatic predominant forms of the disease according to the presence and intensity of individual neuropsychiatric and hepatic signs at the time of diagnosis, as described elsewhere (Litwin et al. 2012).

The control group (CG) consisted of 30 age- and gender-matched healthy volunteers.

All participants with WD underwent brain MRI with a 1.5 T Gyroscan ACS-NT (Philips Medical System, Best, Netherlands). Images were obtained in T1, T2, FLAIR, and T2* sequences, and were subsequently assessed with respect to the presence of changes in 5-mm axial planes. Emphazise was put on regions where most often pathological changes are seen in WD as: lentiform and caudate nuclei, thalami, substantia nigra, red nuclei, pons and cerebellum. Patients were divided into two groups depending on the presence or absence of MRI changes (MRI+ and MRI-, respectively).

Participants from both groups underwent routine ophthalmological examination to rule out ocular pathologies and previous ophthalmological surgery. Exclusion criteria were contraindications to the use of 1 % tropicamide, past ocular surgery, ocular trauma, disease of the anterior and posterior eye segments, and pregnancy. Patients with a refractive error were accepted provided that the error did not exceed ±1.5 diopters. Only one patient (48 year old man) was excluded from our study due to a refractive error of −1.75 D sph as well as myopic retina degeneration, which may have affected the results of our examinations, particularly the electrophysiology. One eye was also excluded from the study due to a history of cataract extraction followed by retinal detachment.

OCT was performed using a Spectralis OCT (Cirrus HD-OT Spectral Domain Technology, Zeiss, Germany) with software version 5.11. Total thickness of the macula (Mth) and of the retinal nerve fiber layer (RNFL) was measured separately. Macular images were manually segmented to determine the thickness of specific layers of the retina at the macula: the ganglion cell and inner plexiform layer complex (GCIP), the inner nuclear layer (INL), the outer plexiform layer (OPL), and the outer nuclear layer plus the photoreceptor layer (ONL + PRL). The thickness of all layers was measured at the thickest point within the perifovea, with the exception of the outer nuclear layer plus the photoreceptor layer, which was measured at the center of the fovea. To maximize accuracy, manual segmentation was performed on black-and-white images. RNFL measurement was performed after manually centering the optic disc. Thickness values for four quadrants (superior, temporal, inferior, and nasal) were automatically generated by the software.

Electrophysiological studies included pattern-reversal visual evoked potentials (PVEP) and flash full-field ERG. Electrophysiological investigations were performed according to standards set by the International Society for Clinical Electrophysiology of Vision using the RetiScan RetiPort gamma plus^2^ system (Roland Consult, Germany) to obtain electrophysiological data during both PVEP assessment and ERG (Odom et al. 2010; McCulloch et al. 2015).

PVEP peak latencies of N75, P100, and N135 waves were measured, as were N75-P100 wave amplitudes. One hundred responses were obtained and averaged for each eye. ERG was performed under both scotopic (dark-adapted) and photopic (light-adapted) conditions. The light stimulus was generated with a whole-field Ganzfeld stimulator. Measurements of responses to both low-intensity (0.01 cd·s·m^−2^; dark-adapted 0.01 ERG) and high-intensity (3.0 cd·s·m^−2^; dark-adapted 3.0 ERG) stimuli were obtained under scotopic conditions; the 3.0 cd·s·m^−2^ stimulus was also evaluated under photopic conditions (light-adapted 3.0 ERG). B-waves were evaluated in response to the low-intensity stimulus under scotopic conditions, while both a- and b-waves were analyzed with the high-intensity stimulus under scotopic and photopic conditions. Amplitude and latency were measured for each wave trace and for the first three oscillatory potentials (O1, O2, and O3).

### Statistical analysis

All individual measurements of thickness (OCT), and electrophysiological parameters (PVEP, ERG) are average values from the left and right eyes. The normality of the distribution of each variable in each group was assessed using the Shapiro-Wilk test. Comparisons of the OCT, PVEP, and ERG results between the MRI+ and MRI- groups and between the MRI- and CG groups were performed using Student’s t test (if variables fulfilled the assumptions of normal distribution) or Mann-Whitney test (if variables did not fulfill the assumptions of normal distribution). Univariate correlations between each of the OCT variables and each of the PVEP, and ERG parameters were evaluated using Spearmann’s correlation coefficients. Average values of the data measured in both eyes (except for one data point due to the exclusion of a single eye as described earlier), were analyzed. Additionally to investigate potential correlation between age and RNFL or Mth Spearman’s rho coefficient was calculated in the control group. The data are presented as means and standard deviation (SD). *P*-values below 0.05 were considered statistically significant.

## Results

The studied cohort consisted of 58 patients (including 34 females; mean age 38.7 years) and was divided into MRI+ (*n* = 39; including 22 females) and MRI- (*n* = 19; including 12 females). The control group (CG) consisted of 30 participants (including 18 females), with a mean age of 39.6 years. Patient characteristics as well as kind of WD treatment are presented in Table 1.

**Table 1:** Patient characteristics [data shown as mean (SD)]

	MRI+ (*n* = 39)	MRI- (*n* = 19)
Age (years)	42.1 (10.71)	31.1 (8.84)
Age at disease onset (years)	31.2 (9.09)	20.3 (8.55)
Age at disease diagnosis (years)	33.3 (10.02)	20.4 (7.03)
Neuropsychiatric form (n) at diagnosis	32	0
Hepatic form (n) at diagnosis	7	19
Neurologic signs and symptoms at ophthalmological assessment (n)	35	1
Duration of disease (years)	10.7 (9.95)	11.1 (10.34)
WD treatment (DPA/ZS)	26/13	6/13

*SD* standard deviation, *n* number of patients, *MRI* magnetic resonance image, *MRI+* patients with pathological findings on MRI, *MRI*- patients without pathological findings on MRI, *DPA-d* penicillamine, *ZS* zinc sulphate

**Fig. 1:**
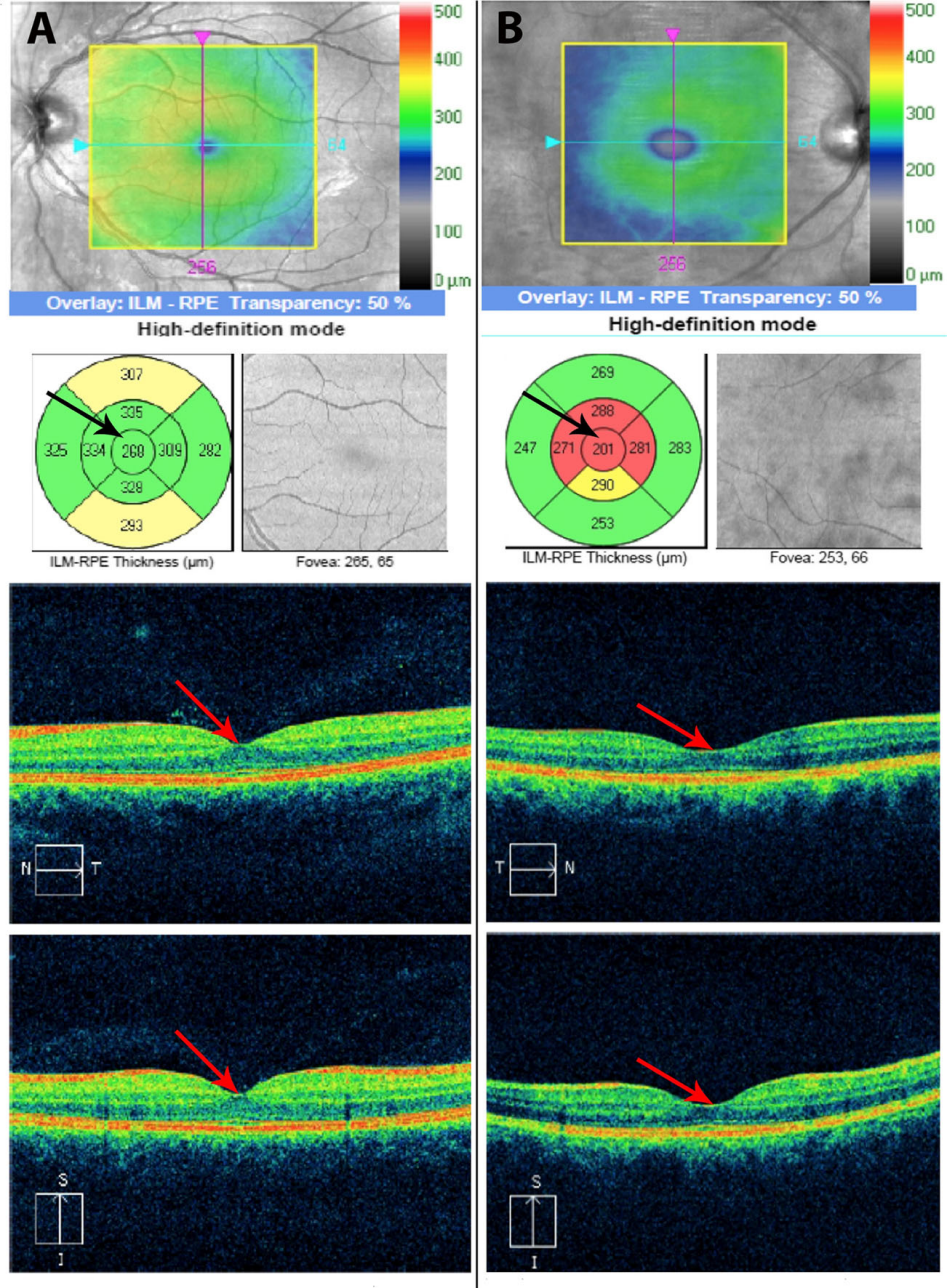
**a** Physiological central macular thickness 268 μm for MRI negative WD patient (arrows show central subfield thickness); **b** Pathological central macular thickness 201 μm for MRI positive WD patient (arrows show central subfield thickness)

**Fig. 2:**
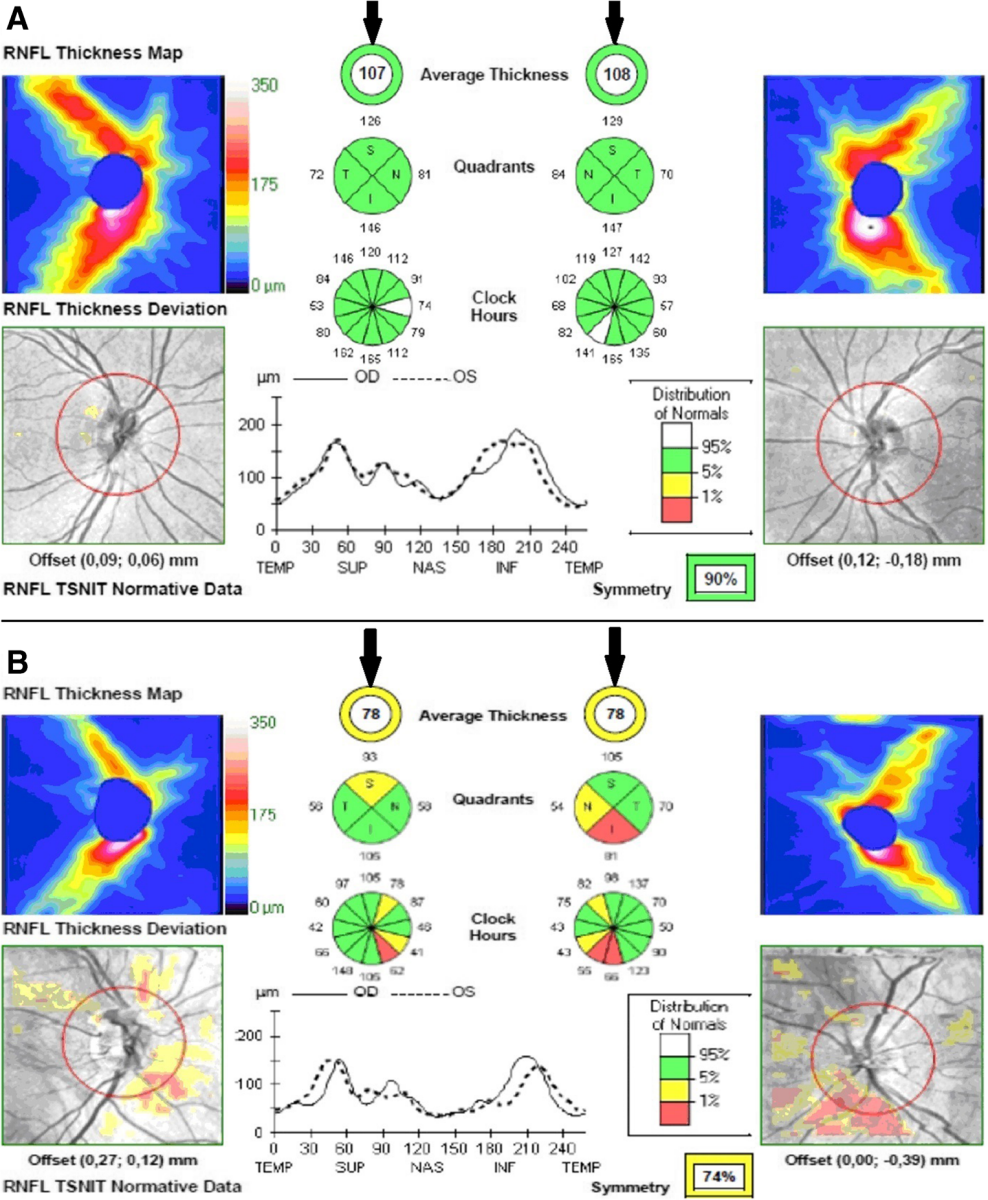
**a** Physiological RNFL for MRI negative WD patient (arrows illustrate 107 μm for right eye and 108 for left eye; **b** Pathological RNFL for MRI positive WD patient (arrows illustrate 78 μm for right eye and 78 for left eye)

Total RNFL (*p* = 0.001) was thinner in the MRI+ group compared to the MRI-group. The RNFL measured in the superior (*p* = 0.024) and temporal (*p* = 0.006) quadrants was also thinner in the MRI+ group. Mth in the MRI+ group was thinner compared to the MRI- group (*p* < 0.001). The manually segmented individual layers were also thinner in the MRI+ group (*p* < 0.05, except for OPL, where *p* = 0.085). No differences were found in the thickness of RNFL and Mth between the MRI- and CG. A detailed breakdown of the OCT results is presented in Table 2, examples of OCT (physiological - for MRI negative patient and pathological for MRI positive patient) are shown in Figs. 1 and 2.

**Table 2:** Results of Ocular Coherent Tomography (OCT) in WD patients with and without MRI pathology

	WD Group						CG and MRI-			
	MRI+ (N = 39)		MRI− (N = 19)		MRI+ vs. MRI-		CG		MRI- vs. CG	
Parameter	Mean	SD	Mean	SD	Difference	P	Mean	SD	Difference	**p**
Retinal nerve fiber layer measurements										
RNFL (μm)	89.13	8.80	96.34	6.41	**−7.21**	**0.001**	96.07	5.05	0.27	0.967
S	109.97	11.04	118.05	12.58	**−8.08**	**0.024**	114.18	11.06	3.87	0.263
T	62.85	9.00	71.66	11.23	**−8.81**	**0.006**	73.85	4.53	−2.19	0.426
I	116.61	13.54	122.32	9.45	−5.71	0.074	120.55	8.27	1.77	0.460
N	71.74	10.28	73.68	8.85	−1.94	0.467	76.27	4.86	−2.59	0.255
Macular thickness measurements										
Mth (μm)	254.82	16.26	273.50	18.57	**−18.68**	**0.001**	279.50	11.04	−6.00	0.103
GCIP	78.61	3.34	83.45	2.14	**−4.84**	**<0.001**	82.95	2.75	0.50	0.483
INL	33.00	2.33	36.92	1.59	**−3.92**	**<0.001**	37.28	1.07	−0.36	0.364
OPL	30.56	1.45	31.16	1.05	−0.60	0.085	30.75	1.28	0.41	0.399
ONL + PRL	118.50	3.92	121.21	4.42	**−2.71**	**0.031**	119.15	2.99	2.06	0.122

*MRI* magnetic resonance image, *MRI+* patients with pathological findings on MRI, *MRI*- patients without pathological findings on MRI, *CG* control group, *RNFL* retinal nerve fibre layer, *S* superior part, *T* temporal part, *I* inferior part, *N* nasal part, *Mth* total macular thickness, *GCIP* ganglion cell and inner plexiform layer, *INL* inner nuclear layer, *OPL* outer plexiform layer, *ONL + PRL* outer nuclear layer and photoreceptor layer, *SD* standard deviation, Statistically significant differences are represented in bold

Latencies of all the measured PVEP components were prolonged in the MRI+ group. N75 latency (*p* < 0.001), P100 latency (*p* < 0.001), and N135 latency (*p* = 0.019) were all prolonged when compared to the MRI- group. Contrary to the effect seen for latencies, the N75-P100 amplitude was not different between both groups. Only N135 latency was prolonged in the MRI- group when compared to the CG (*p* < 0.001), no other differences were observed between the MRI- group and CG. Detailed PVEP results are presented in Table 3.

**Table 3:** Visual evoked potential latencies (ms) and amplitudes (μV) in Wilson Disease with and without pathological changes in brain MRI

	WD Group						CG and MRI-			
	MRI+ (N = 39)		MRI− (N = 19)		MRI+ vs. MRI-		CG		MRI- vs. CG	
Parameter	Mean	SD	Mean	SD	Difference	p	Mean	SD	Difference	p
N75 (ms)	78.40	3.25	75.05	2.62	**3.35**	**<0.001**	74.73	1.73	0.32	0.643
P100 (ms)	114.07	7.06	106.44	4.09	**7.63**	**<0.001**	105.79	2.26	0.65	0.902
N135 (ms)	148.40	3.78	145.58	3.82	**2.82**	**0.019**	138.97	3.73	**6.61**	**<0.001**
Amplitude N75 - P100 (μV)	9.67	2.09	10.20	2.33	0.53	0.411	9.30	1.62	0.90	0.152

*MRI* magnetic resonance image, *MRI+* patients with pathological findings on MRI, *MRI-* patients without pathological findings on MRI, *CG* control group, *N75* N75 wave latency, *P100* P100 wave latency, *N135* N135 wave latency, *N75-P100 amplitude* amplitude between peaks of N75 and P100 waves, *SD* standard deviation, *ms* milliseconds, *μV* microvolts. Statistically significant differences are represented in bold

In general, ERG latencies were prolonged in the MRI+ group while amplitudes were diminished, when compared to the MRI- group.

Dark-adapted 0.01 ERG demonstrated increased b-wave latency (*p* < 0.001) and reduced b-wave amplitude (*p* = 0.033) in the MRI+ group compared to the MRI- group (Table 4).

**Table 4:** Electroretinography latencies and amplitudes in Wilson disease with and without pathological changes in brain MRI

	WD Group						CG and MRI-			
	MRI+ (N = 39)		MRI− (N = 19)		MRI+ vs. MRI-		CG		MRI- vs. CG	
Parameter	Mean	SD	Mean	SD	Difference	p	Mean	SD	Difference	p
Dark-adapted 0.01										
sc b lat. (ms)	87.96	5.50	81.41	3.29	**6.55**	**<0.001**	78.42	5.49	2.99	0.023
sc b amp. (μV)	204.53	56.62	242.00	59.21	**−37.47**	**0.033**	242.83	56.73	−0.83	0.926
Dark-adapted 3.0										
sc max a lat. (ms)	21.82	1.62	19.77	1.70	**2.05**	**<0.001**	20.99	1.24	**−1.22**	**0.004**
sc max a amp. (μV)	238.38	74.64	271.32	81.98	−32.94	0.117	343.37	35.50	**−72.05**	**0.001**
sc max b lat. (ms)	45.09	2.99	41.72	2.67	**3.37**	**<0.001**	40.54	3.43	1.18	0.377
sc max b amp. (μV)	444.85	118.10	492.50	133.60	−43.65	0.232	562.60	76.99	−70.1	0.058
O1 lat. (ms)	20.03	2.23	18.39	0.87	**1.64**	**0.001**	18.21	0.46	0.18	0.418
O1 amp. (μV)	22.19	7.65	28.69	8.43	**− 6.5**	**0.008**	30.66	5.31	−1.97	0.371
O2 lat. (ms)	25.76	1.43	24.56	0.90	**1.2**	**0.001**	24.73	0.93	−0.17	0.542
O2 amp. (μV)	52.27	23.40	68.13	29.84	**- 15.86**	**0.049**	95.50	24.16	**−27.37**	**0.006**
O3 lat. (ms)	32.50	1.33	31.73	1.03	**0.77**	**0.030**	31.49	0.90	0.24	0.417
O3 amp. (μV)	23.02	11.65	27.88	14.30	−4.86	0.377	49.92	17.68	**−22.04**	**<0.001**
Light-adapted 3.0										
pht a lat. (ms)	16.23	1.12	15.46	0.70	**0.77**	**0.001**	15.03	0.60	**0.43**	**0.018**
pht a amp. (μV)	43.48	14.34	56.75	14.07	**−13.27**	**0.002**	56.08	8.46	0.67	0.853
pht b lat. (ms)	31.34	0.96	30.46	1.16	**0.85**	**0.008**	30.45	1.06	0.01	0.983
pht b amp. (μV)	167.66	40.45	203.95	53.80	**−36.29**	**0.015**	234.47	41.01	**−30.52**	**0.043**

*MRI* magnetic resonance image; *MRI+* patients with pathological findings on MRI, *MRI-* patients without pathological findings on MRI, *CG* control group, *sc b lat.* b wave latency, *sc b amp* b-wave amplitude. *Lat.* latency, *amp.* amplitude, *O1, O2, O3* first, second and third oscillatory potential respectively, *SD* standard deviation, *ms* milliseconds, *μV* microvolts. Statistically significant differences are represented in bold

**Fig. 3:**
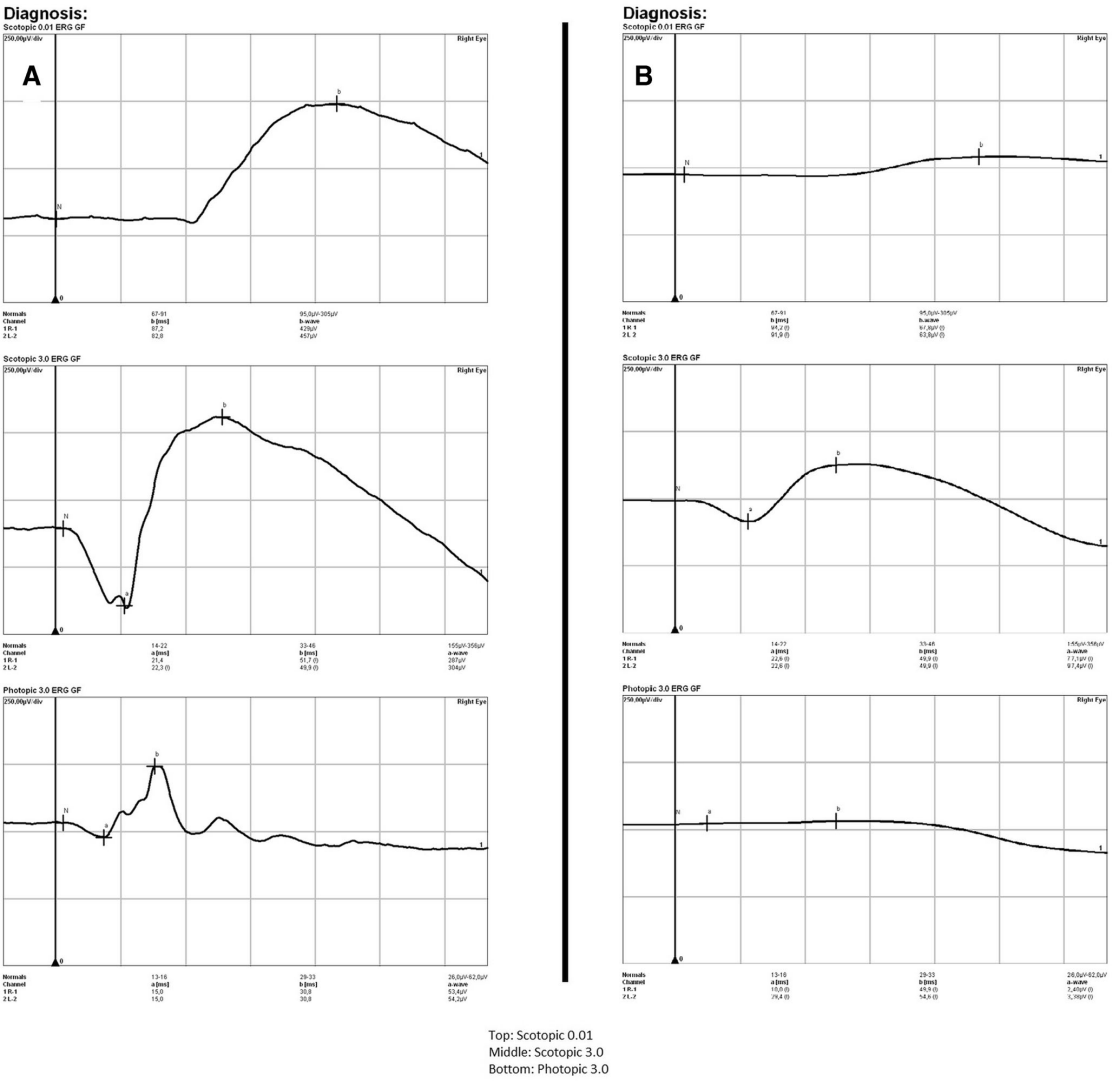
**a** Physiological ERG for MRI negative WD patient; **b** Pathological ERG for MRI positive patient (decreased amplitudes of a-wave and b-wave)

**Fig. 4:**
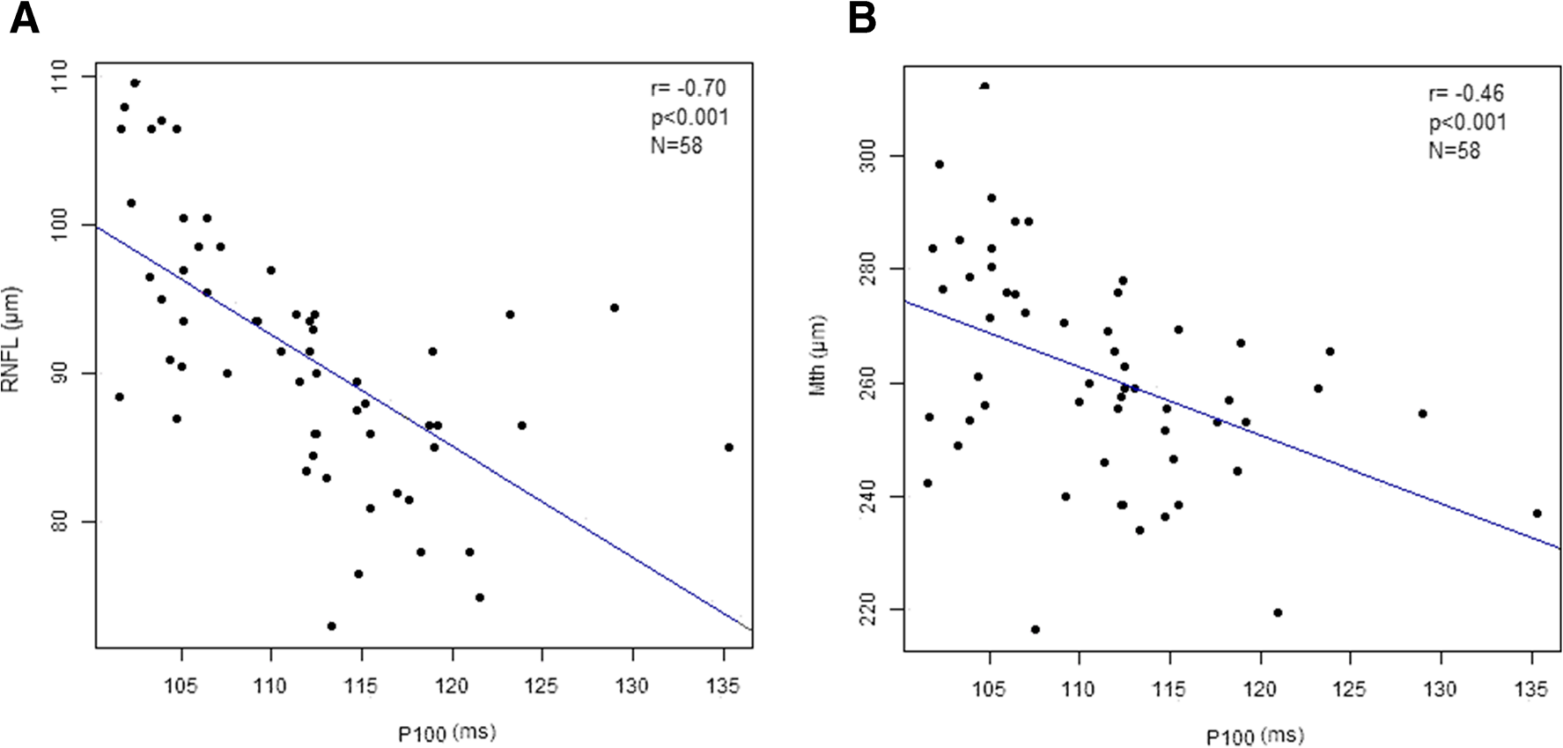
**a** Correlation between P100 latency and RNFL thickness in patients with WD. **b** Correlation between P100 latency and total macular thickness in patients with Wilson’s disease

Dark-adapted 3.0 ERG showed a prolonged latency of both the a-wave (*p* < 0.001) and b-wave (*p* < 0.001) in the MRI+ group compared to the MRI-group. No differences however, were observed for the amplitude values between these groups. When compared to the CG though, the MRI- group showed prolonged a-wave latency (*p* = 0.017), and reduced a-wave amplitude (*p* < 0.001). No differences in the b-waves parameters were seen (Table 4). In the dark-adapted 3.0 ERG prolonged latencies of the O1 (*p* = 0.001), O2 (*p* = 0.001), and O3 (*p* = 0.030) oscillatory potentials were also observed in the MRI+ group, in addition to reduced amplitudes of the O1 (*p* = 0.008) and O2 (*p* = 0.049) potentials. The O2 (*p* = 0.001) and O3 (*p* < 0.001) amplitudes were also reduced in the MRI- group when compared to the CG (Table 4). In the light-adapted 3.0 ERG test, the latencies of the a-wave (*p* = 0.001) and b-wave (*p* = 0.008) were also prolonged in the MRI+ group with respect to the MRI- group. The respective amplitudes were diminished (a-wave: *p* = 0.002; b-wave: *p* = 0.015). The a-wave latency was prolonged (*p* = 0.015), and the b-wave amplitude diminished (*p* = 0.020) in the MRI- group in comparison to the CG. Detailed ERG results are presented in Table 4, while examples of ERG (physiological for MRI negative patient and pathological for MRI positive patient) are shown in Fig. 3. Additionally, in the entire cohort, the P100 latency measured on PVEP correlated negatively with the OCT measurements of RNFL (R = −0.70, *p* < 0.001; Fig. 4a) and Mth (R = −0.46, *p* < 0.001; Fig. 4b). Finally, there was no correlation between age and RNFL (R = −0,28, *p* = 0139) or Mth (R = −0,25, *p* = 0186) in CG.

## Discussion

Our findings confirm morphological and functional status of retinal and visual pathways in patients with WD, which are more pronounced in individuals with brain MRI pathology. While Albrecht et al. demonstrated OCT and electrophysiology changes in WD patients, their findings did not consider clinical variations between their patients, treating them as a uniform group. Furthermore, their electrophysiology examinations only tested VEP, and did not include ERG testing in order to investigate the retina. Our cohort of 58 patients was larger, homogenous (no asymptomatic individuals) and the performed electrophysiological examinations encompassed both VEP and ERG, which allowed us to evaluate WD patients’ retinas from a physiological point of view. We focused mainly on morphological and physiological changes present in patients with pathological lesions evident on brain MRI compared with WD patients without visible lesions and with age- and gender-matched healthy controls. Although the mean age variance between the MRI+ and MRI- groups was 11 years, numerous authors as well as our analysis state that age is not an influential factor for OCT, RNFL thickness and electrophysiology (Sabates et al. 2011; Feuer et al. 2011; Freud et al. 2011; Kanamori et al. 2003), Mailankody et al. 2015.

In terms of morphology, considerable thinning of the macula and RNFL occurred in the MRI+ group (Table 2). The RNFL is mainly composed of unmyelinated axons of ganglion cells located in the ganglion cell layer. Measuring RFNL thickness by the OCT method gives us an objective estimation of the integrity of unmyelinated axons. Retinal architecture, which can also be assessed by OCT, estimates neuronal integrity of the retina. The largest contributor to reduced Mth in both absolute and relative terms came from the GCIP and the inner nuclear layer. The inner nuclear layer consists of tightly packed cell bodies of bipolar, horizontal, and amacrine cells (Kaufman and Alm 2003). Interestingly, the only assessed layer that did not significantly differ between the groups was the OPL. This is in line with the work of (Albrecht et al. 2012). The OPL is the junction between the photoreceptor and the bipolar and horizontal cells. The outer two-thirds of the OPL consist of photoreceptor axons and Muller cell processes (Henle fiber layer), while the rest of the OPL (dendritic OPL) consists of dendrites from the horizontal and bipolar cells of the of INL, as well as Muller cell processes (Kaufman and Alm 2003). As the OPL morphology was unchanged in both groups of patients, it may be less affected by copper toxicity than other layers.

There were no abnormal morphological findings in MRI- patients compared to the CG, which may suggest that retinal thinning occurs in more advanced stages of WD or predominantly in patients with more severe brain pathology that is visible on MRI.

There are various clinical forms of WD, and suffering from WD does not automatically affect macular and RNFL thickness. In our study, the MRI- group of WD patients did not appear to differ from the CG with respect to RNFL thickness, suggesting that our finding of RNFL thinning is specifically related to brain changes evident on MRI. Our observation of thinner RNFLs in MRI+ patients supports previous observations that chronic copper-related degeneration affects unmyelinated fibers (Valenti 2011; Garcia-Martin et al. 2014). However, while hepatic forms of WD should theoretically lead to smaller OCT changes, more advances stages of the disease (without neurological signs but with MRI pathology), even in this form, may bring OCT abnormalities.

Interestingly, retrograde thinning of the RNFL was previously proposed to be an early marker of neurodegeneration (Valenti 2011). It is considered to be one of the earliest signs of Alzheimer’s disease, occurring even prior to hippocampal damage that impacts memory (Valenti 2011). We speculate that this finding may reflect differential susceptibility of the RNFL to different mechanisms of neurodegeneration. Patients with multiple sclerosis also exhibit thinning of the retina versus healthy controls. However, this effect is most pronounced in patients with a history of optic neuritis in the course of their disease (Garcia-Martin et al. 2014).

Functional impairment of retinal and visual pathways, was demonstrated by differences in PEVP and ERG between MRI+ and MRI- subjects in the present investigation, confirms the multifocal character of WD.

Various ERG waves are known to originate in morphologically distinct regions of the retina. Alterations in the a-wave reflect changes in the receptor cell layer, those in the b-wave correspond to bipolar and horizontal cell pathology, and oscillatory potentials are thought to originate from amacrine cells.

Many neurodegenerative diseases (e.g. multiple sclerosis) may initially show functional changes detected only by electrophysiological methods, without evident morphological or clinical abnormalities (Simo et al. 2008). Upon comparing the results of the MRI- patients to the control group, only electrophysiological changes (a- wave in sc max, pht a- and b- waves and OP) were discovered, while the OCT was unchanged (Tables 3 and 4). This may point to functional abnormalities, particularly in the receptor and amacrine cells, which could confirm that electrophysiological changes may occur earlier than morphological disorders. Differences between the CG and the MRI- group, as well as between the MRI- and MRI+ groups, could be regarded as the earliest electrophysiological manifestations of WD. These changes include the amplitudes of oscillatory potentials and a-wave amplitude under 3.0 dark-adapted conditions. ERG sc max a-wave amplitude and latency, as well as OP changes, point towards initial visual system damage in the MRI- group. Oscillatory potentials that are most likely generated by amacrine cells, appear to reflect the activity of the negative feedback exerted by amacrine cells towards bipolar and ganglion cells. Importantly, impairment of oscillatory potentials is a very sensitive marker that reflects very early pathology related to oxidative stress; for example, reductions in oscillatory potentials occur in the initial stages of diabetic retinopathy (Langwinska-Wosko 1993). The observed changes are sensitive markers of initial disorders of the retina therefore may be present at early stages of WD when brain pathology is not evident.

Other differences between the MRI- group and the CG observed here can be regarded as intermediate changes in which differences occur across the spectrum of WD (photopic a-wave latency and b-wave amplitude). The remaining parameters only differed between the MRI+ and MRI- groups, indicating that they are specific correlates of changes on MRI that are typical for advanced WD.

From our data, we cautiously conclude that amacrine cells are among the first cells to be affected by WD. Furthermore, cones may be more vulnerable than rods (earlier findings in photopic 3.0 ERG) as our electrophysiology examinations, showed most changes to pht a latency and pht b amplitude – characteristic of cone function. Meanwhile, rod functions are portrayed by sc b, which were normal in MRI- patients.

Whether ERG alterations reflect a conduction defect within cells, or a defect in synaptic transmission, remains to be elucidated. It seems likely that the observed pathology is predominantly intracellular in origin, as copper accumulates within the cell in WD (Zischka and Lichtmannegger 2014; Dusek et al. 2015).

General electrophysiological status of the visual pathway also seems to reflect changes to the central nervous system visible on MRI, corroborating the notion of continuity between the brain and the retina. Increased latency without concomitant decreased amplitude of PVEP waves clearly points to a conduction defect in visual pathways. Two mechanisms have been postulated to account for this effect. The first hypothesis predicts that copper deposits directly affect conduction, while the second identifies secondary demyelination as the culprit (Albrecht et al. 2012). The latter explanation seems particularly attractive because similar electrophysiological changes were observed in multiple sclerosis (Garcia-Martin et al. 2014). It also provides an elegant explanation of why latencies are affected but not amplitudes: demyelination is fundamentally a problem of conduction velocity. Interestingly, while changes visible on MRI correlated with all PVEP wave latencies in the present investigation, we also detected a significant difference in N135 latency between the MRI- group and the CG, suggesting that increased latency of the N135 wave may be an early electrophysiological manifestation of WD progression. This increased latency increased as MRI changes became more visible, making it a potentially useful marker of WD severity. Unfortunately, the N135 wave is regarded to be the least stable of PVEP waves.

Prolonged latency of the P100 wave is considered to be the most robust and clinically relevant marker of severe impairment of visual-pathway function. This conclusion is consistent with our observation that P100 waves were only delayed in the MRI+ group (reflecting advanced pathology), as well as with the strong correlations with retinal thinning detected here. RNFL and macula thinning would therefore result in conduction delays within visual pathways.

Existing research suggests that neurodegeneration in WD may be reflected in measurable retinal and PVEP findings (Topcu et al. 2002; Albrecht et al. 2012). Significant retinal thinning was previously observed in WD patients via OCT (Albrecht et al. 2012). Furthermore, children with WD exhibit prolonged latencies of all visual evoked potential waves compared to healthy controls (Albrecht et al. 2012). Similarly, significant changes are evident on ERG, particularly under dark-adapted conditions, and are reversible with treatment (Satishchandra and Ravishankar Naik 2000), highlighting the usefulness of retinal investigations during treatment monitoring. Pre-symptomatic siblings of WD patients are also more likely to exhibit changes in visual evoked potential curves (Topcu et al. 2002), suggesting that these data would be useful when screening at-risk populations.

Our work provides evidence that WD patients with pathological changes on brain MRI tend to perform worse on various ophthalmological tests than WD patients with normal MRI. We also identified electrophysiological pathology in patients with no lesions visible on MRI. In general, at least some electrophysiological findings preceded the onset of changes evident on OCT and MRI, and morphological retinal changes were generally absent unless changes on MRI had already emerged. Correlations between OCT and electrophysiological changes across our population point toward a singular underlying pathology.

Regarding the limitations of our study, we note that while we conducted detailed ophthalmological investigations, we only concentrated on the correlation of these data with MRI-detectable brain pathology not with clinical presentation. We also did not perform analysis of retinal changes with clinical form of WD (hepatic vs. neuropsychiatric), however most our MRI positive patients had neurological signs on diagnosis (36/58; 62 %). Such study should be done before starting treatment. Our patients were treated for several years, and their initial symptoms changed, so we concentrated only on brain pathology. Only prospective long term study on patients who just starts anti-copper therapy, may provide some information about role of copper intoxication on retinal pathology and function in WD and to test if ophthalmological tests could be used as potential marker of treatment effectiveness.

Summarized, our study confirmed that OCT, ERG, and PVEP are potentially valuable clinical tools for the investigation of organ injury in WD and provides a foundation for investigating further correlations between retinal pathology and clinical characteristics of WD patients, as well as patient response to anti-copper therapy.
